# Exercise-Induced Circulating Hematopoietic Stem and Progenitor Cells in Well-Trained Subjects

**DOI:** 10.3389/fphys.2020.00308

**Published:** 2020-05-07

**Authors:** Julia M. Kröpfl, Fernando G. Beltrami, Hans-Jürgen Gruber, Ingeborg Stelzer, Christina M. Spengler

**Affiliations:** ^1^Exercise Physiology Lab, Institute of Human Movement Sciences and Sport, ETH Zurich, Zurich, Switzerland; ^2^Clinical Institute of Medical and Chemical Laboratory Diagnostics, Medical University of Graz, Graz, Austria; ^3^Institute of Medical and Chemical Laboratory Diagnostics, LKH Hochsteiermark, Leoben, Austria; ^4^Zurich Center for Integrative Human Physiology, University of Zurich, Zurich, Switzerland

**Keywords:** high-intensity interval exercise, hematopoietic stem and progenitor cells, oxidative stress, cell self-renewal, median fluorescent intensity

## Abstract

It has been proposed that exercise-induced systemic oxidative stress increases circulating hematopoietic stem and progenitor cell (HPC) number in active participants, while HPC clonogenicity is reduced post-exercise. However, HPCs could be protected against exercise-induced reactive oxygen species in a trained state. Therefore, we characterized the acute exercise-induced HPC profile of well-trained participants including cell number, clonogenicity, and clearance. Twenty-one healthy, well-trained participants—12 runners, 9 cyclists; age 30.0 (4.3) years—performed a strenuous acute exercise session consisting of 4 bouts of 4-min high-intensity with 3-min low-intensity in-between, which is known to elicit oxidative stress. Average power/speed of intense phases was 85% of the peak achieved in a previous incremental test. Before and 10 min after exercise, CD34+/45dim cell number and clonogenicity, total oxidative (TOC), and antioxidative (TAC) capacities, as well as CD31 expression on detected HPCs were investigated. TOC significantly decreased from 0.093 (0.059) nmol/l to 0.083 (0.052) nmol/l post-exercise (*p* = 0.044). Although HPC proportions significantly declined below baseline (from 0.103 (0.037)% to 0.079 (0.028)% of mononuclear cells, *p* < 0.001), HPC concentrations increased post-exercise [2.10 (0.75) cells/μl to 2.46 (0.98) cells/μl, *p* = 0.002] without interaction between exercise modalities, while HPC clonogenicity was unaffected. Relating HPC concentrations and clonogenicity to exercise session specific (anti-) oxidative parameters, no association was found. CD31 median fluorescent intensity expression on detected HPCs was diminished post-exercise [from 1,675.9 (661.0) to 1,527.1 (558.9), *p* = 0.023] and positively correlated with TOC (*r*_*rm*_ = 0.60, *p* = 0.005). These results suggest that acute exercise-reduced oxidative stress influences HPC clearance but not mobilization in well-trained participants. Furthermore, a well-trained state protected HPCs’ clonogenicity from post-exercise decline.

## Introduction

Circulating hematopoietic stem and progenitor cells (HPCs) are precursor cells of the immune system and support blood and tissue regeneration throughout a lifetime, especially if circulation is kept youthful by physical exercise and training (Stelzer et al., 2010). In the untrained, acute exercise induces oxidative stress by an increased turnover rate of the mitochondrial transport chain due to higher energetic demands. In well-trained participants, exercise-induced (anti-) oxidative behavior is less clear, depending on which oxidative stress marker(s), enzymatic or non-enzymatic antioxidants, are investigated (Finaud et al., 2006). Acute exercise also influences circulating HPC number and clonogenicity (Emmons et al., 2016a). Different mechanisms responsible for exercise-induced HPC mobilization have recently been discussed (e.g., sympathetic stress, shear forces, or inflammation) in addition to possible mechanisms for exercise-induced HPC clearance (e.g., by chemoattractant production in tissue, respective receptor expression on HPCs, and subsequent cell homing) (Emmons et al., 2016a). The importance of oxidative stress in this “push/pull mechanism” shortly after acute exercise pushing HPCs from bone marrow to circulation and then pulling them into surrounding tissues or bone marrow, however, is inconclusive.

Systemic oxidative stress increases HPC number shortly (10 min) after acute exercise in active participants, while at the same time HPC clonogenicity is reduced (Schraml et al., 2009; Kroepfl et al., 2012). However, HPCs are suggested to be desensitized to exercise-induced oxidative stress by increasing inherent levels of protection with training, termed exercise hormesis (De Lisio and Parise, 2013). Each individual exercise session in a training program causes mild oxidative stress, triggering an adaptive response to diminish the stress of subsequent exercise sessions (Radak et al., 2008). Since HPC quantity and differentiation along the lineage are increased in the mouse bone marrow after training, exercise-induced oxidative stress might have an attenuated effect on HPC number, and clonogenicity in a trained state. A study showing this attenuated or even lacking influence of exercise-induced oxidative stress on precursor cells of the hematopoietic system in trained participants, however, is lacking in literature.

The role of oxidative stress in exercise-induced HPC clearance has yet not been identified but could be related to inflammatory signaling (Glennon-Alty et al., 2018). The expression of CD31, a cell adhesion molecule with immune modulating properties, has been shown to be upregulated during inflammation and is necessary for leukocyte (Muller, 2015) and HPC transendothelial migration (Yong et al., 1998). In naive *T* cells, a downregulation of surface CD31 expression was even related to homeostatic cell proliferation (Kohler and Thiel, 2009). Therefore, we hypothesized that exercise-induced oxidative stress could be responsible for the modulation of CD31 expression on HPCs altering their clearance in well-trained participants.

As mentioned, however, other mechanisms apart from oxidative stress have also been proposed to play a role in HPC mobilization with exercise. In this regard, exercise-induced oxidative stress was shown to be comparable between running and cycling in recreationally active individuals (Kouvelioti et al., 2019), but blood viscosity during cycling was shear rate dependent, while this effect was not apparent during running (Nader et al., 2018). Thus, comparing the effects of these two exercise modalities on HPC mobilization might assist in controlling for the potential impact of differences in exercise-induced shear stress on HPCs. Furthermore, running could result in a higher damage of the endothelial cell layer and musculature than cycling (Uhlemann et al., 2014) and might therefore differently impact HPC mobilization and clearance. As such, we included both running and cycling in the study design.

The specific aims of this study were therefore 1. to assess changes in HPC number and clonogenicity following an acute exercise session of running or cycling known to induce oxidative stress; 2. to evaluate a possible exercise-induced change in CD31 expression on detected HPCs; and 3. to elucidate a possible relationship between exercise-induced oxidative stress and HPC number, clonogenicity, as well as CD31 expression in well-trained participants.

We hypothesized that exercise-induced oxidative stress would not influence HPC number and clonogenicity but would decrease CD31 expression on HPCs independent of the exercise modality.

## Methods

### Participant Recruitment

Participants were consecutively recruited, and thoroughly informed about all procedures. The study conformed to the standards set by the Declaration of Helsinki.

### Subject Characteristics and Study Design

Twenty-one healthy, well-trained participants (male: *n* = 19, female: *n* = 2) participated in the study. Participants were 30.0 (4.3) years, had a body mass index of 22.4 (1.8) kg⋅m^–2^, 14.9 (4.2)% body fat, 10.8 (3.2) kg fat mass, 61.7 (7.2) kg fat-free mass, and a V̇O_2_peak of 60.4 (5.5) ml⋅min^–1^⋅kg^–1^, which met all inclusion/exclusion criteria for the study. All participants underwent an acute exercise session in one of two possible exercise modalities and were experienced runners (*n* = 12) or cyclists (*n* = 9).

### Exercise Trial

A traditional high-intensity interval training (HIIT) session was used, which is known to elicit oxidative stress post-exercise (Kruger et al., 2016). The acute exercise consisted of four bouts of 4-min high-intensity cycling/running interspersed with 3 min of low-intensity exercise. The first bout was preceded by a 5-min warm-up with 3 min at 100 W/1 km⋅h^–1^ and 2 min at 50% of the difference between the first stage and the first bout, and followed by 3 min of cooldown. Average speed or power of intense phases was 85% of maximal speed or power from a previous incremental test (100 W + 20 W⋅min^–1^ or 10 km⋅h^–1^ + 1 km⋅h^–1^⋅min^–1^), depending on the exercise modality. Work was matched between the two exercise modalities.

### Body Composition Assessment

Fat mass, fat-free mass, and percent body fat were measured by dual-energy X-ray absorptiometry (GE Healthcare, Luna iDXA).

### Hematological Analysis and Mononuclear Cell Isolation

At baseline and 10 min after exercise cessation, 23 ml of venous blood was withdrawn from the cubital vein. Serum was isolated as recommended (1,500 *g*, 10 min, and 23°C) and kept frozen at −80°C until analysis. Three hundred microliters of whole blood was kept for hematological analysis (ADVIA 2120i, Siemens, Zurich, Switzerland), and the remaining volume was subjected to a standard Ficoll gradient centrifugation (Histopaque, Sigma-Aldrich, Switzerland) within 2 h after blood withdrawal in order to isolate peripheral blood mononuclear cells (MNCs) for HPC analyses. HPC number was assessed by flow cytometry in all participants. In a subgroup of participants, HPC clonogenicity was investigated by colony-forming unit assays in cell culture.

### Flow Cytometry

**FIGURE 1:**
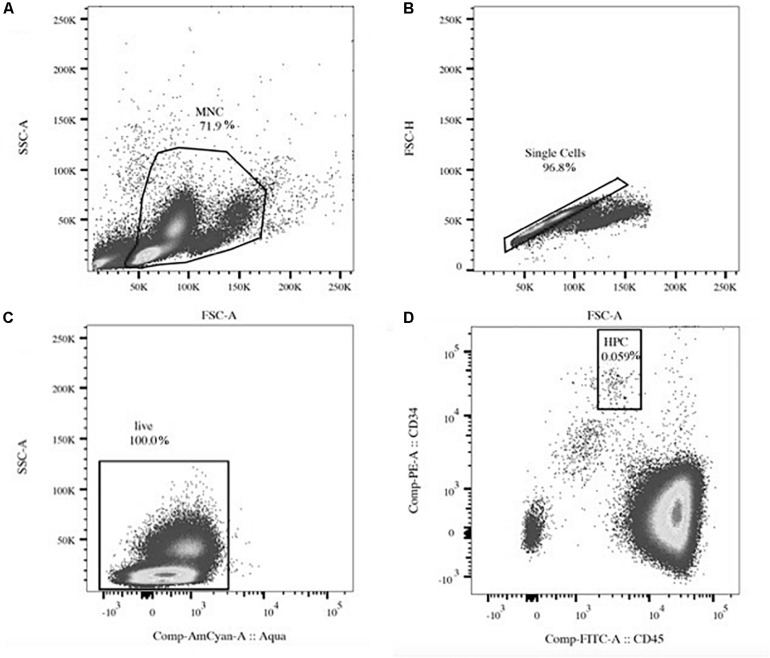
Exemplary flow cytometry gating. Light scatter gating of the mononuclear cell (MNC, **A**) population and doublet exclusion (single cells, **B**). Fluorescent gating of live cells **(C)** and the hematopoietic stem and progenitor cell (HPC) population identified as CD34+/CD45dim within the MNC gate **(D)**. Percent numbers indicate cell amount relative to the parent population.

Flow cytometry sample preparation and gating strategy were based on published protocols (Duda et al., 2007; Bellows et al., 2011), but final analysis was done with slight modifications as follows: one to 1.2 million MNCs were stained in phosphate buffered saline including fetal bovine serum (2%) and ethylenediaminetetraacetic acid (0.4%) with CD34-phycoerythrin, CD45-fluorescein-isothiocynate (Thermo Fisher Scientific, Zurich, Switzerland), and CD31-allophycocyanin-Cy7 (Lucerna-Chem AG, Lucerne, Switzerland) >30 min on ice. After washing (1,500 rpm, 5 min, and 10°C), MNCs were incubated with a fixable Aqua stain (Thermo Fisher Scientific, Zurich, Switzerland) for live/dead discrimination for >10 min. After a last wash, the stain was fixed in 4% paraformaldehyde (Fisher Scientific, Ontario, Canada) in PBS and analyzed on a FACS Canto II device (BD Biosciences, Allschwil, Switzerland)—equipment of the flow cytometry facility, University of Zurich, Switzerland—using a FACSDiva software within 2 h of fixation. Analysis was done in duplicate. Three-color flow cytometry was performed with compensated fluorescent parameters (BD^TM^ CompBead, BD Biosciences, Allschwil, Switzerland) including appropriate Fluorescence Minus One controls. The main acquisition gate was established based on forward and side scatter characteristics including lymphocytes and monocytes (MNCs), but excluding debris. After doublet exclusion and gating for live cells, circulating HPCs (CD34+/CD45dim) were analyzed within at least 200,000 MNCs (Figure 1). Final flow cytometry data were investigated with a separate analysis tool (FlowJo, LLC, OR, United States). Relative HPC content (proportion) was given as %MNCs. Estimates of HPC concentrations were given as cells/μl calculated by multiplying respective total HPC proportions by the absolute number of MNCs in peripheral blood measured by a standard hematology analyzer (Kropfl et al., 2019). In addition, median fluorescent intensity (MFI) of CD31 expressed on all detected HPCs per sample was extracted (Niemiro et al., 2018).

### Colony-Forming Unit Assays

Colony-forming unit-granulocyte macrophage (CFU-GM) assays were done as previously described (Kroepfl et al., 2012; Kropfl et al., 2014). Briefly, 200,000 MNCs/ml were seeded in methylcellulose medium without erythropoietin (Methocult H4534; StemCell Technologies, Vancouver, Canada) and incubated at 37°C (5% CO_2_, >95% humidity). On the eighth day of cell culture, colonies consisting of at least 40 cells were scored, and provided the number of early hematopoietic progenitor cell colonies (primary CFU-GM, early clonogenicity) of each participant. Next, up to 90 primary CFU-GM colonies were individually plucked from the methylcellulose culture medium and transferred to individual wells. After another 2 weeks of incubation, each well was scored for the number of CFU-GM colonies (>40 cells, secondary CFU-GM). The secondary replating capacity (prolonged clonogenicity) measured as area under the curve (AUC) is known to be associated with the self-renewal ability of myeloid progenitor cells (Gordon et al., 1998).

### Total (Anti-) Oxidative Capacities

Total oxidative capacity (TOC) and total antioxidative capacity (TAC) were determined in serum using the Labor Diagnostika Nord (Nordhorn, Germany) assays according to the manufacturer’s instructions. The determination of both TOC and TAC is based on the reaction of peroxides with peroxidase followed by a color reaction of the chromogenic substrate tetramethylbenzidine. Serum samples need to be assayed by subtraction of initial absorption. Quantification is achieved by serial dilutions of a standard peroxide or antioxidant solution.

The oxidative index (OI) was calculated as the TOC/TAC ratio (Tatzber et al., 2003).

### Statistics

Data are represented as mean (standard deviation, SD). *A priori* sample size calculation (Kroepfl et al., 2012) revealed a necessary sample size of *n* = 21 in order to detect a significant correlation between exercise-induced oxidative stress and HPC number with a power >0.8 using a linear bivariate regression model, one-tailed, α = 0.05, ρ = 0.5, SD_*oxidative stress*_ = 0.03 μmol/l, and SD_*HPCs*_ = 162.3 cells/ml. A repeated-measures ANOVA (repeated effect: time; group effect: exercise modality) was used for analysis of blood parameters. *Post-hoc* tests were done if needed. Between-group comparisons were performed for participant characteristics and performance variables by unpaired *t*-tests or Mann–Whitney *U* tests depending on variable distribution. Pearson (coefficient *r*) or repeated measures correlation (coefficient *r*_*rm*_) analyses were used to determine the relationship between variables. Unlike simple correlation, repeated measures correlation analysis does not violate the assumption of independence of observations and tends to have much greater statistical power in the case of a repeated measures design. The slopes that are displayed identify the best fit for all intraindividual associations (Bakdash and Marusich, 2017). Calculations were done with R3.3.0 using the available package “rmcorr.” In addition, G^∗^Power3.1.7, IBM SPSS Statistics25, and GraphPad Prism8 were used for analysis and visualizations. A *p*-value < 0.050 was significant.

## Results

### Exercise Trial

The exercise session specific power was 83.9 (1.7)% of peak power output (PPO) from a previous incremental test. Runners’ and cyclists’ subject and performance characteristics were as follows, respectively, age: 30.3 (3.8) years, 29.6 (5.1) years; body mass index: 22.1 (1.6) kg⋅m^–2^, 22.8 (2.1) kg⋅m^–2^; PPO during the incremental test: 17.2 (1.1) km⋅h^–1^, 414.8 (29.5) W; average power during the intensive bouts of the acute exercise: 83.9 (2.0)% PPO, 83.8 (1.3)% PPO; average oxygen intake during the intensive bouts of the acute exercise: 3,667.5 (518.4) ml⋅min^–1^, 3,989.8 (315.1) ml⋅min^–1^.

### Total (Anti-) Oxidative Capacities

**FIGURE 2:**
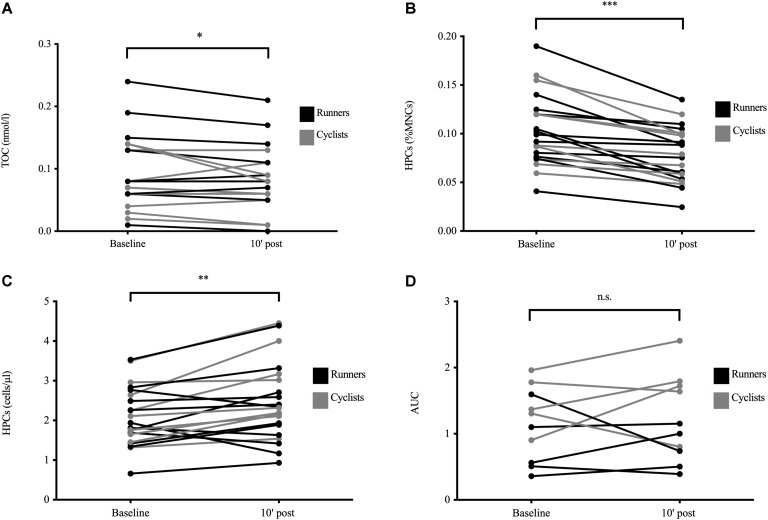
Exercise-induced blood and serum parameters in well-trained participants. Total oxidative capacity (TOC, *n* = 20, **A**), circulating hematopoietic stem and progenitor cell (HPC) proportions as percentage of acquired mononuclear cells (MNCs) assessed by flow cytometry (*n* = 21, **B**), HPC concentrations calculated by multiplying HPC proportions with the respective MNC numbers from the hemocytogram (*n* = 21, **C**), and secondary colony formation defined by the cells’ prolonged clonogenicity *in vitro* (*n* = 10, **D**) at baseline and 10 min after exercise cessation. Color differences indicate the two different exercise modalities. Significant differences between time points are indicated by *p < 0.050, **p < 0.010, and ***p < 0.001; n.s., non-significant. Analysis was done by mixed ANOVA with one repeated factor (time) and one group factor (exercise modality).

There was a significant main effect of time for TOC (*p* = 0.040) significantly decreasing from 0.093 (0.059) nmol/l to 0.083 (0.052) nmol/l shortly after exercise cessation (Figure 2A). No interaction between exercise modalities could be detected. Neither TAC nor OI showed any significant main or interaction effects (all *p* > 0.050).

### Progenitor Cell Proportions and Concentrations and According Parameter Relationships

There were significant main effects of time with HPC proportions significantly decreasing from 0.103 (0.037)% to 0.079 (0.028)% MNCs (*p* < 0.001; Figure 2B) and HPC concentrations increasing from 2.10 (0.75) cells/μl to 2.46 (0.98) cells/μl after exercise (*p* = 0.002; Figure 2C), with no interaction between exercise modalities. Baseline HPC proportions were significantly associated with the respective exercise-induced HPC loss (*r* = −0.66, *p* = 0.001, *n* = 21, data not shown), but not correlated to the proportions’ percentage change (*p* > 0.05). There were no significant relationships between HPC proportions, HPC concentrations, or their exercise-induced changes and the respective TOC, TAC, and OI concentrations (all *p* > 0.05). Estimates of HPC baseline concentrations were not significantly related to any body composition variable (all *p* > 0.05).

### Progenitor Cell Early and Prolonged Clonogenicity and According Parameter Relationships

Both primary CFU-GM (early clonogenicity) and secondary CFU-GM (prolonged clonogenicity; Figure 2D) did not significantly differ after exercise from baseline values nor between exercise modalities (main and interaction effects, *p* > 0.050). There were no significant correlations between primary or secondary CFU-GM and TOC or TAC concentrations, OI, or any body composition variable (all *p* > 0.050).

### CD31 Expression on Hematopoietic Stem and Progenitor Cells and According Parameter Relationships

**FIGURE 3:**
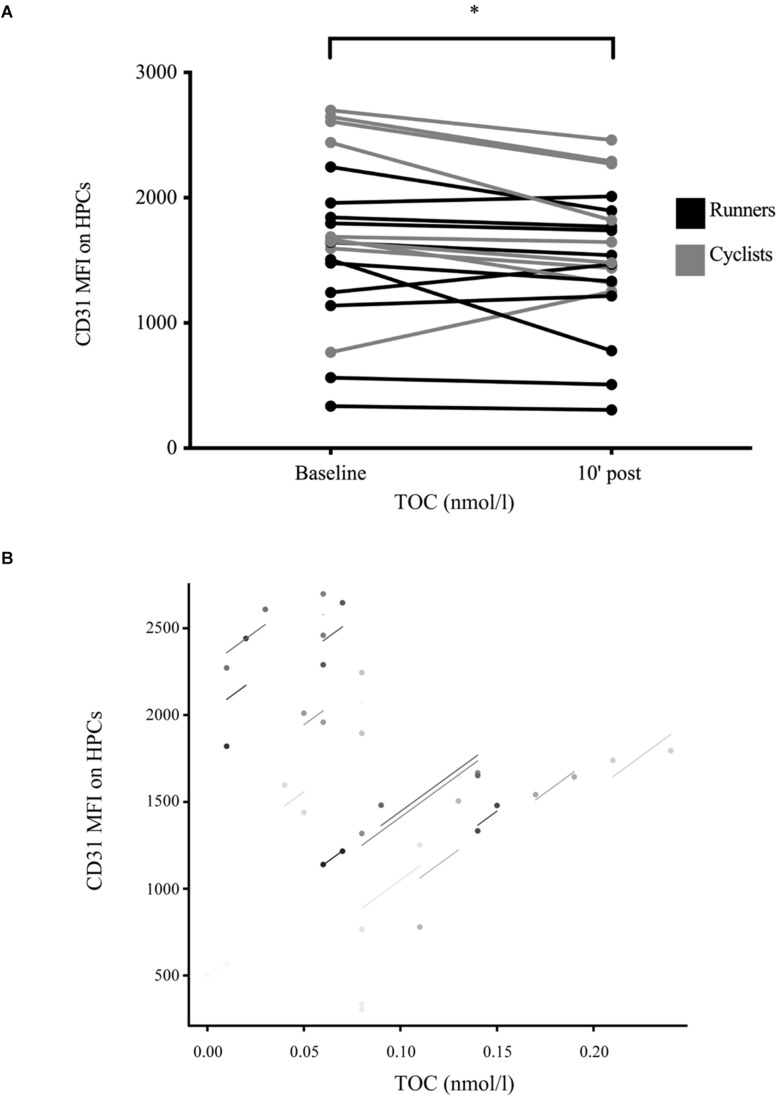
Exercise-induced CD31 median fluorescent intensity expression on hematopoietic stem and progenitor cells in well-trained participants. **(A)** Median fluorescent intensity (MFI) expression of the cell surface protein CD31 on detected circulating hematopoietic stem and progenitor cells (HPCs) was assessed at baseline and 10 min after exercise cessation. Results were significantly decreased post-exercise. Color differences indicate the two different exercise modalities. *n* = 20. **(B)** Total oxidative capacity (TOC) was positively associated with CD31 MFI expression on detected HPCs (*r*_*rm*_ = 0.60, *p* = 0.005, and *n* = 19). Significant differences are indicated by *p < 0.050 and were assessed by mixed ANOVA with one repeated factor (time) and one group factor (exercise modality). Parameter relation was addressed by repeated measures correlation analysis.

CD31 MFI expressed on HPCs showed a significant main effect of time with MFI significantly decreasing from 1,675.9 (661.0) to 1,527.1 (558.9) after exercise (*p* = 0.020) without interaction between exercise modalities (Figure 3A). There was a significant correlation between CD31 MFI to TOC concentrations (*r*_*rm*_ = 0.60, *p* = 0.005, *n* = 19; Figure 3B), but no correlation to TAC or OI concentrations (both *p* > 0.050).

### Blood Cell Counts and According Parameter Relationships

A summary of descriptive statistics of blood cell counts can be found in Table 1. Shortly after exercise, white blood cell counts (*p* < 0.001), red blood cell counts (*p* = 0.005), hematocrit (*p* < 0.001), hemoglobin (*p* < 0.001), mean corpuscular volume (*p* = 0.018), monocytes (MONO, *p* = 0.007), lymphocytes (LYM, *p* < 0.001), neutrophils (NEU, *p* < 0.001), and lymphocyte proportions (%LYM, *p* < 0.001) significantly increased. At the same time, neutrophil proportions (%NEU, *p* < 0.001) and basophil proportions (%BASO, *p* = 0.014) significantly decreased. Significant interaction effects were visible for HCT (*p* = 0.030), MONO (*p* = 0.028), BASO (*p* = 0.023), and %BASO (*p* = 0.043). There were no significant correlations between LYM, %LYM, MONO, %MONO, NEU, and %NEU with TOC, TAC, or OI (all *p* > 0.050).

**TABLE 1:** Blood cell counts.

**Blood Collection**	**Baseline HIIT Runner**	**10’post HIIT Runner**	**Baseline HIIT Cyclist**	**10’post HIIT Cyclist**
WBC, 10^9^/l	4.7(1.1)	6.2(1.7)	5.3(0.6)	7.3(1.3)***
RBC, 10^12^/l	4.68(0.49)	4.85(0.40)	4.90(0.12)	5.10(0.13)**
Hct, %	43.8(3.1)	44.6(3.2)	44.8(1.7)	47.0(2.0)***^§§§^
Hgb, g/dl	15.0(1.41)	15.3(1.2)	15.4(0.5)	16.1(0.5)***
MCV, fl	91.9(3.4)	92.3(3.8)	91.4(2.4)	92.2(2.8)*
Lymphocytes, 10^3^/μl	1.61(0.43)	2.53(0.74)	1.0(0.45)	2.86(0.54)***
Monocytes, 10^3^/μl	0.37(0.20)	0.41(0.21)	0.37(0.07)	0.66(0.35)**^§^
Neutrophils, 10^3^/μl	2.41(0.95)	2.89(1.05)	2.79(0.50)	3.26(0.71)***
Basophils, 10^3^/μl	0.04(0.02)	0.04(0.01)	0.04(0.02)	0.05(0.01)^§^
Lymphocytes, %	35.4(8.8)	41.2(7.7)	34.1(6.8)	39.5(4.2)***
Monocytes, %	8.1(3.8)	6.8(3.3)	7.0(0.7)	8.9(3.9)
Neutrophils, %	50.3(10.1)	46.1(8.5)	53.1(7.9)	45.3(7.6)***
Basophils, %	0.91(0.39)	0.61(0.18)^§^	0.71(0.27)	0.68(0.20)*

Values are reported as mean (SD), *n* = 21. HIIT, traditional high-intensity interval protocol; WBC, white blood cell count; RBC, red blood cell count; Hct, hematocrit; Hgb, hemoglobin; main effects of time are indicated as follows: ***p < 0.001, **p < 0.010, and *p < 0.050; significant differences between time points within the same exercise modality are indicated as follows: ^§^*p* < 0.050, ^§§§^*p* < 0.001.

## Discussion

This is the first study to show that acute strenuous exercise-induced HPC increase shortly after exercise cessation is not linked to the oxidative or antioxidative system in well-trained participants. However, our data hint at a possible exercise-attenuated HPC clearing mechanism by diminished CD31 surface expression on detected HPCs that was positively associated with circulating TOC concentrations. In addition, early HPC clonogenicity and prolonged HPC clonogenicity were not affected by the acute strenuous exercise.

Acute exercise immediately upregulates both (anti-) oxidative systems in untrained participants (Jamurtas et al., 2018), and exercise-induced oxidative stress is significantly related to the change in HPCs (Kroepfl et al., 2012). Exercise-induced responses in well-trained participants, however, are less clear (Finaud et al., 2006; De Lisio and Parise, 2013). Based on previous literature, the exercise session used was designed to induce an acute increase in systemic oxidative stress (Kruger et al., 2016). Our results showed, however, an acute exercise-induced decrease of TOC 10 min post-exercise with no effects on TAC or OI possibly due to a decrease of oxidative stress production during exercise or an increase in the antioxidant system efficiency (Finaud et al., 2006). The downregulation of TOC was not associated with changes in HPCs, which suggests that HPCs are not influenced by systemic oxidative stress shortly after exercise cessation in a trained state (De Lisio and Parise, 2013). In addition, both running and cycling induced a comparable HPC increase, suggesting that neither shear rate-dependent blood viscosity during cycling (Nader et al., 2018) nor a higher endothelial cell layer damage during running (Uhlemann et al., 2014) could have been mainly responsible for HPC change. Nonetheless, a certain influence of exercise-induced shear stress or endothelial damage on HPC change cannot be excluded with our study design. The post-exercise increase in HPCs was possibly triggered by a mechanism other than oxidative or shear stress, the most likely being increased sympathetic activity (Agha et al., 2017).

Notably, HPC proportions showed a stronger exercise-induced decline when HPC proportions at baseline were high, suggesting a threshold to which acute strenuous exercise can decrease HPCs within the MNC population. On average, HPC proportions declined by 18.5% post-exercise. One study so far reported that 6 weeks of endurance exercise training reduced both HPC proportions and concentrations post-exercise (Niemiro et al., 2018). Our study extends these findings by showing that a single acute exercise session had a comparable effect on HPC proportions but simultaneously increased HPC concentrations in well-trained participants. It is important to note that HPC concentrations are calculated estimates from mature immune cell numbers (Kropfl et al., 2019), and that acute exercise—in contrast to exercise training—induced lymphocytosis and monocytosis and therefore also a post-exercise HPC concentration increase. Moreover, an animal study also found an exercise training-induced increase of HPC proportions in the bone marrow, indicating increased HPC maintenance and preservation (Baker et al., 2011). This could also be the reason for the lower HPC baseline concentrations found in athletes compared to sedentary controls (D’ascenzi et al., 2015).

Interestingly, both early clonogenicity and prolonged clonogenicity were unchanged post-exercise and not related to the exercise-induced reduction in oxidative stress. This is a different outcome than seen in less trained participants where HPC colony-forming capacity significantly declined shortly after exercise cessation (Kroepfl et al., 2012) by an adrenergic mechanism (Kropfl et al., 2014). An improved HPC clonogenicity in bone marrow was already reported in exercise-trained mice (Baker et al., 2011) possibly due to increased endocrine signaling from skeletal muscle. The unchanged outcome in HPC clonogenicity post-exercise in our study is possibly another sign for exercise hormesis in well-trained participants.

Exercise training-induced HPC clearance in human participants was found to depend on chemotactic gradients and chemokine receptor expression on HPCs, where a higher receptor expression was associated with more cell homing and tissue infiltration (Niemiro et al., 2018). Acute exercise-induced mature immune cell clearance was explained by an enhanced cell receptor expression 24 h post-exercise in human participants (O’carroll et al., 2019). An animal model, however, also suggested HPCs to home to peripheral tissues shortly after acute exercise cessation (Emmons et al., 2016b). We build upon these findings by showing that acute exercise-reduced oxidative stress decreased CD31 surface expression on HPCs shortly after exercise cessation in well-trained participants, possibly indicating attenuated HPC clearance. The process of leukocyte and HPC clearance from the circulation, termed transendothelial migration (TEM), was shown to be regulated among others by CD31 expression on the leukocyte, HPC, and the endothelial cell border (Yong et al., 1998; Muller, 2015). During TEM, CD31 expressed on both the leukocyte and the endothelium bind, and a disruption of this interaction would arrest leukocytes on the apical surface of the epithelium (Muller, 2015). We suggest that this could be the same for HPCs stimulated by exercise-reduced oxidative stress, since anti-CD31 incubation did not prevent CD34+cells from endothelial adhesion, but from TEM (Yong et al., 1998). In addition, mice with bone marrow precursor cells lacking CD31 showed an increase in HPCs in the peripheral blood explained by a reduced ability to migrate from blood to the bone marrow vascular niche (Ross et al., 2008). This way, the HPC pool in circulation could be maintained.

### Limitations

The lack of a less trained control group for comparison is a limitation of the present study. However, results of oxidative stress as well as HPC number and clonogenicity were compared against the results of our own previously published work in active participants (Kroepfl et al., 2012). Baseline results of CD31 expression on HPCs were significantly lower than unpublished preliminary results from active participants [8016.2 (1184.6), *p* < 0.001, *n* = 5] investigated with the same flow cytometry panel and gating procedure. This indicated higher exercise-induced HPC clearance by CD31 upregulation in less trained participants.

The lack of determining the expression of the homing chemokine (C-X-C motif) receptor 4 on HPCs and protein concentrations of its ligand CXCL-12 in plasma are additional limitations of the study design. This information would have added to the knowledge of exercise hormesis in trained participants, especially since recent studies did not find any significant exercise-induced CXCR-4 expression change on HPCs (Ross et al., 2018) or mature angiogenic T-cells (O’carroll et al., 2019) in active subjects. However, based on previous data from our own work (Kammerer et al., 2020), and others (Theiss et al., 2008), we would not expect any change in CXCR-4 mRNA expression in circulating MNCs or an increase in circulating CD34+/CXCR-4 cell number under acute exercise in normoxic conditions. Therefore, the focus of this study was solely on HPC clearance *via* transendothelial migration by CD31 expression on HPCs and not by the CXCR-4/CXCL-12 axis. In addition, HPC clonogenicity only explains the potential for cell self-renewal in the peripheral blood—a transwell-migration assay would have been needed to fully elucidate HPC function.

This study was not designed to detect any sex-specific difference in HPC mobilization by physical exercise in trained subjects. According to an *a priori* power analysis based on data from exercise-induced CD34+/VEGFR2+ cells in active subjects (Shill et al., 2016) a sample size of *n* = 36 would have been necessary to answer this research question. Furthermore, we were not excluding participants on the base of sex—the male/female ratio of participants (*n* = 19/2) happened by chance.

## Conclusion

Although acute strenuous exercise significantly increased HPC concentrations in well-trained participants shortly after exercise cessation, exercise-induced oxidative stress was not the respective trigger possibly due to participants’ elevated training status. Rather, the exercise-induced reduction in systemic oxidative stress seemed to be involved in attenuating HPC clearance from the circulation over the endothelium to surrounding tissues or bone marrow by diminishing the expression of the cell adhesion molecule CD31 on HPC surfaces. Interestingly, the HPC profile of well-trained participants further showed that HPC clonogenicity did not decline post-exercise, possibly another sign for exercise hormesis. These results support and extend the already existing knowledge of the positive effects of a trained state on precursor cells of the immune system and add to the evidence that acute exercise does not suppress but rather challenges/boosts the immune system shortly after exercise cessation (Campbell and Turner, 2018), since HPCs are maintained in circulation.

## Data Availability Statement

All datasets generated for this study are included in the article/supplementary material.

## Ethics Statement

The study involving human participants was reviewed and approved by Cantonal Ethics Committee, Stampfenbachstrasse 121, 8090 Zurich, Switzerland (BASEC 2017-00128). The participants provided their written informed consent to participate in this study.

## Author Contributions

The experiments were performed partly at the Exercise Physiology Lab (ETH Zurich, Switzerland) and the Division of Hematology (University Hospital Zurich, Switzerland). JK, FB, and CS were responsible for the conception or design of the work. JK, FB, H-JG, and IS drafted the work or revised it critically for important intellectual content and performed data acquisition and/or analysis. All authors were responsible for interpretation of data for the work. In addition, all authors approved the final version of the manuscript, agreed to be accountable for all aspects of the work in ensuring that questions related to the accuracy or integrity of any part of the work were appropriately investigated and resolved. All persons designated as authors qualify for authorship, and all those who qualify for authorship are listed.

## Conflict of Interest

The authors declare that the research was conducted in the absence of any commercial or financial relationships that could be construed as a potential conflict of interest.
